# Identification of the Related Substances in Ampicillin Capsule by Rapid Resolution Liquid Chromatography Coupled with Electrospray Ionization Tandem Mass Spectrometry

**DOI:** 10.1155/2014/397492

**Published:** 2014-11-04

**Authors:** Lei Zhang, Xian Long Cheng, Yang Liu, Miao Liang, Honghuan Dong, Beiran Lv, Wenning Yang, Zhiqiang Luo, Mingmin Tang

**Affiliations:** ^1^School of Chinese Materia Medica, Beijing University of Chinese Medicine, No. 6 Zhonghuan South Road, Wangjing, Chaoyang District, Beijing 100102, China; ^2^Institute for the Control of Traditional Chinese Medicine and Ethnic Medicine, National Institutes for Food and Drug Control, State Food and Drug Administration, 2 Tiantan Xili, Beijing 100050, China

## Abstract

Rapid Resolution Liquid Chromatography coupled with Electrospray Ionization Tandem Mass Spectrometry (RRLC-ESI-MS^n^) was used to separate and identify related substances in ampicillin capsule. The fragmentation behaviors of related substances were used to identify their chemical structures. Finally, a total of 13 related substances in ampicillin capsule were identified, including four identified components for the first time and three groups of isomers on the basis of the exact mass, fragmentation behaviors, retention time, and chemical structures in the literature. This study avoided time-consuming and complex chemosynthesis of related substances of ampicillin and the results could be useful for the quality control of ampicillin capsule to guarantee its safety in clinic. In the meantime, it provided a good example for the rapid identification of chemical structures of related substances of drugs.

## 1. Introduction

Ampicillin is an important semisynthetic *β*-lactam antibiotic and it is still widely used nowadays because of its good efficacy in urinary tract infections, respiratory infections, and other diseases caused by germs and bacteria. In recent years, the requirement of quality control for related substances in chemicals became stricter no matter in structure confirmation or content limitation. Ampicillin was especially degradable in presence of aqueous solution or humid storage environment, which would lead to the formation of a variety of degradation products [1]. These related substances (the related substances previously reported were shown in Table 1) would have a great influence on the quality of the products and clinical medication safety.

Although there has been much research on the related substances of ampicillin [2, 3], it is not completely explicit so far. To ensure the clinical safety and meet the new requirement of related substances in chemicals [4], it is still necessary to conduct further studies to develop a rapid and efficient method to describe in more detail the related substances of ampicillin capsule.

Many analytical methods including high-performance liquid chromatography (HPLC) [1, 5], high-performance capillary electrophoresis (HPCE) [6], high-performance liquid chromatography-atmospheric pressure chemical ionization mass (HPLC-APCI-MS) [7], and high-performance liquid chromatography-electrospray mass spectrometry (HPLC-ESI-MS) [8, 9] had been utilized for the analysis of ampicillin. Among these methods, LC-ESI-MS had been shown to be a powerful technique for the analysis of ampicillin and its related substances due to its excellent ability in separation and identification.

In this paper, a simple, rapid, and sensitive Rapid Resolution Liquid Chromatography coupled with Electrospray Ionization Tandem Mass Spectrometry (RRLC-ESI-MS^n^) method was established for the identification of the related substances in ampicillin capsule. The result suggested that this technique could facilitate rapid and accurate identification of related substances in ampicillin capsule.

## 2. Experimental

### 2.1. Chemicals and Materials

Methanol (HPLC grade) was purchased from Fisher Scientific (Pittsburgh, PA, USA). Formic acid (HPLC grade) was obtained from Acros Organics (Geel, Belgium). Deionized water was further purified with a Milli-Q water system (Bedford, Massachusetts, USA). Ampicillin capsule was purchased from DAVA Pharmaceuticals. Inc. (Huntsville, AL, USA).

The chromatographic separation was performed with an Agilent 1200 series Rapid Resolution Liquid Chromatography system (Agilent Technologies, USA), equipped with a binary pump, a microvacuum degasser, a high-performance autosampler, a column compartment, a diode array detector, and a MS detector. The samples were separated on a 1.8 *μ*m Agilent Zorbax XDB-C_18_ column (50 mm × 4.6 mm) at a flow rate of 0.4 mL*·*min^−1^. The mobile phases consisted of 0.1% formic acid solution (A) and methanol (B). The optimized RRLC elution conditions were as follows: 0–2 min, 10% B; 2–10 min, 10–20% B; 10–20 min, 20–50% B; 20–25 min, 50% B; 25-25.1 min, 50–10% B; 25.1–30 min, 10% B. DAD spectra were acquired over a scan range of 190–400 nm. The sample volume injected was 1 *μ*L. Agilent 6320 mass spectrometer with an Agilent ChemStation to control and process the data was performed with the ESI source in positive ion mode. The vaporizer temperature was maintained at 300°C. The temperature of the drying gas was set at 350°C. The flow rate of the drying gas and the pressure of the nebulizing gas were set at 12 L*·*min^−1^ and 35 psi, respectively. The capillary voltage was kept at 3.5 × 10^3^ V. The mass spectrometer scanned from a mass-to-charge ratio (*m*/*z*) 100–900.

### 2.2. Preparation of Sample

The contents of ampicillin capsule (equivalent to 10 mg Ampicillin) were dissolved in 10 mL methanol and then filtered through a 0.22 *μ*m syringe filter. And an aliquot (1 *μ*L) of the filtrate was subjected to RRLC-ESI-MS^n^ for analysis.

## 3. Results and Discussion

### 3.1. Investigation of the Fragmentation Patterns of Ampicillin

**Figure 1:**
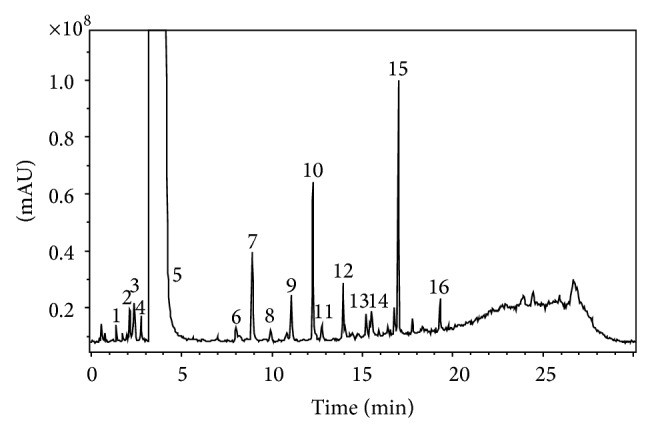
The total ion chromatography of ampicillin capsule (DAVA). The peaks were numbered according to their retention time.

**Figure 2:**
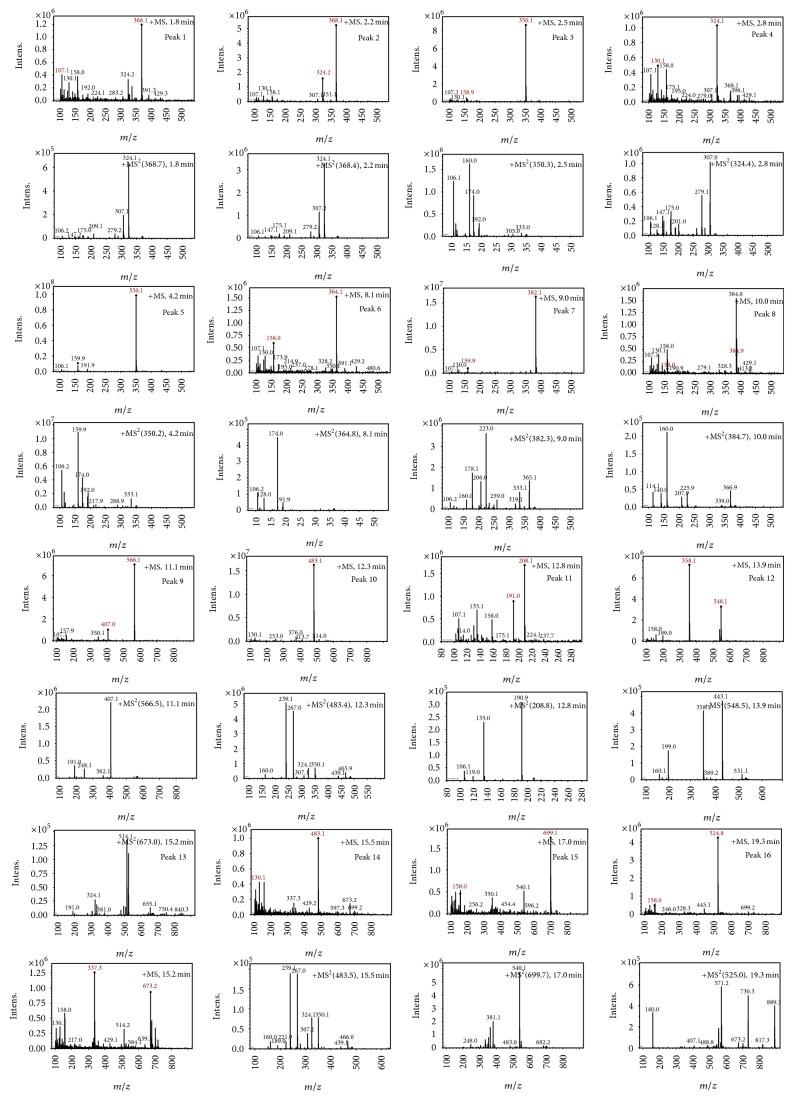
The mass spectrum of 16 chemicals in ampicillin capsule.

It was necessary to study the characterization of the mass spectra of the parent drug to identify the molecular structure of the related substances in ampicillin capsule. Identifications were based on the fact that the related substances of ampicillin usually contain structural fragments and analogous cleavage characteristic of the parent drug. Structural information and fragmentation mechanisms had been deduced from ions in the mass and collision spectra. This knowledge was useful in the analysis and identification of related substances in ampicillin capsule. We utilized knowledge of characteristic fragment ions of ampicillin and its related substances to identify their structures. Figures 1 and 2 showed the detailed total ion chromatography (TIC) and mass spectrum of ampicillin and its related substances, respectively.

**Figure 3:**
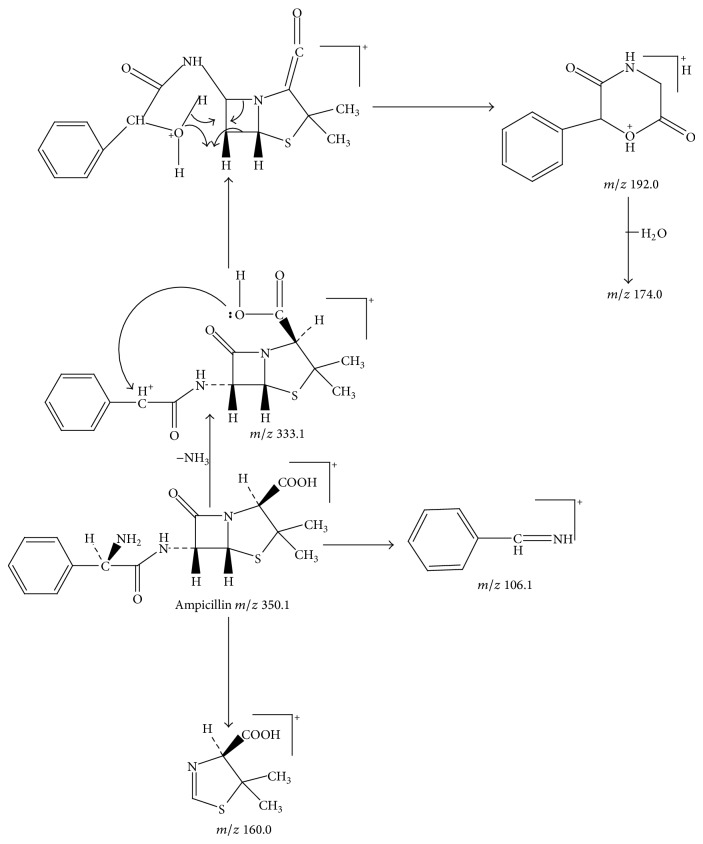
Proposed fragmentation pathways and characteristic ions of protonated ampicillin (*m*/*z* 350).

Ampicillin yielded an abundant ion in the ESI mass spectrum at *m*/*z* 350.1. The ESI mass spectrum of this ion (*m*/*z* 350.1) was shown in Figure 2. The fragment ions at *m*/*z* 106.2 and 160.0 were reported to arise from the benzylamine group and the thiazolidine ring. The fragment ion at *m*/*z* 192.0 was proposed to arise as a result of losing a −NH_2_ group at the benzylamine side chain followed by an oxygen rearrangement and cleavage of the *β*-lactam ring. The fragment ion at *m*/*z* 174.0 could be attributed to the loss of H_2_O from the fragment at *m*/*z* 192.0, but it might arise from other pathways. The proposed fragmentation pathways of ampicillin were shown in Figure 3.

### 3.2. Identification of the Known Related Substances in Ampicillin Capsule

This part of the investigation focused on the characterization of the ESI-MS properties of the parent drug and its known related substances.  Table 2 showed the chromatographic and mass spectral characteristics of the detected related substances in ampicillin capsule.

**Figure 4:**
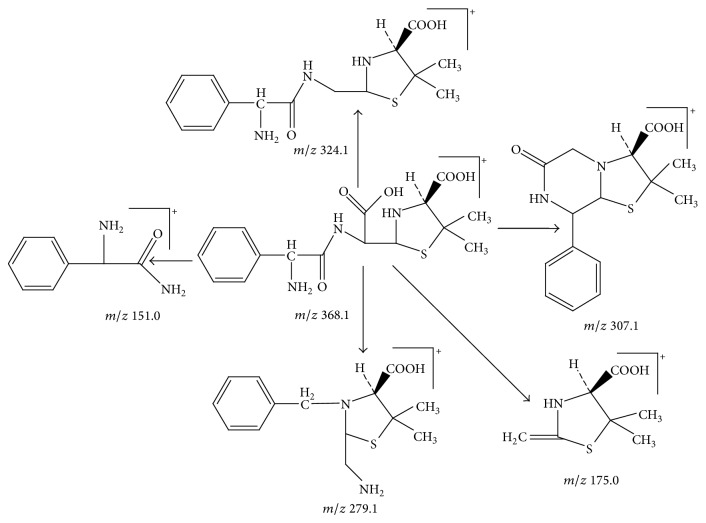
Proposed fragmentation pathway for the fragmentation ions of ampicilloic acid (*m*/*z* 368).

Peak 1 and Peak 2 showed the same MS data. All of them produced protonated quasimolecular ion at *m*/*z* 368.1 [M + H]^+^, major ions at *m*/*z* 324.1, 307.1, 279.2, and 175.1 in ESI^+^ mode. Based on diagnostic ions (*m*/*z* 324.1, 307.1, and 175.1) and comparison with the published literature of known related substances of ampicillin [10], Peak 1 and Peak 2 were identified as isomers of ampicilloic acid. (5S, 6R) or (5R, 6R) ampicilloic acids were the two groups of ampicilloic acid isomers which were reported to be the metabolites and degradation products of ampicillin [1]. According to the retention behavior in reversed-phase chromatography of Peak 1 and Peak 2 and the related literature [1], Peak 1 and Peak 2 were tentatively identified as (5S, 6R) ampicilloic acid and (5R, 6R) ampicilloic acid, respectively.  Figure 4 showed the proposed MS fragmentation pathway for the fragmentation ions of ampicilloic acid.

**Figure 5:**
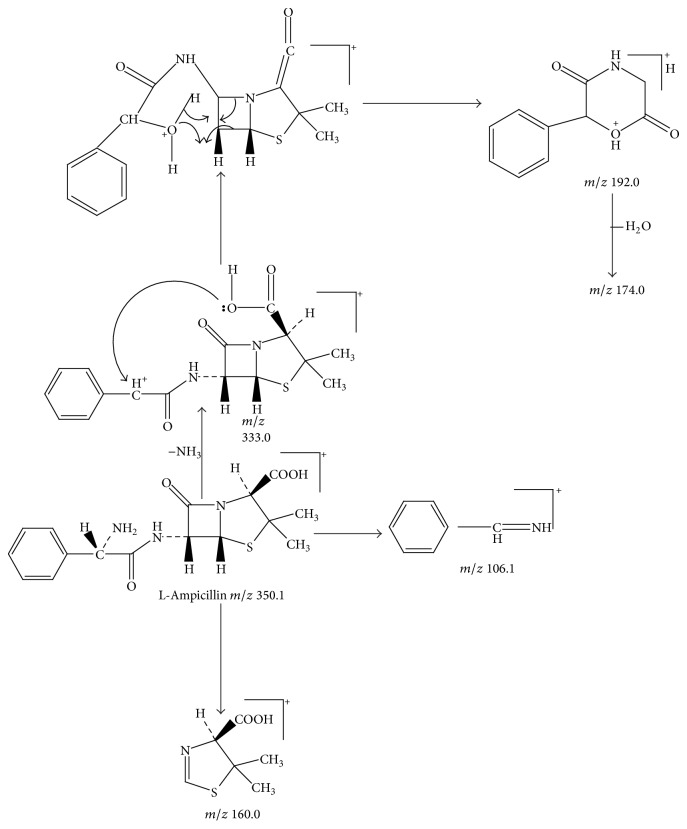
Proposed fragmentation pathways and characteristic ions of protonated L-ampicillin (*m*/*z* 350).

Peak 3 produced a protonated molecular ion at *m*/*z* 350.1 [M + H]^+^, fragment ions at *m*/*z* 333.0 [M − NH_3_]^+^, 192.0, 174.0, 160.0, and 106.1 in ESI^+^ mode. Peak 5 was clearly identified as ampicillin based on comparison of its retention time and mass spectrometric data with reference standards [8]. Peak 3 showed the same fragment ions, fragmentation pattern, and characteristic ions as Peak 5. Therefore, we could conclude that Peak 3 was an isomer of Peak 5. Considering that Peak 3 had a much shorter retention time than Peak 5, and with the comparison of related substances reported in the literature [1], Peak 3 was tentatively identified as L-ampicillin.  Figure 5 showed the proposed MS fragmentation pathway for the fragmentation ions of L-ampicillin.

**Figure 6:**
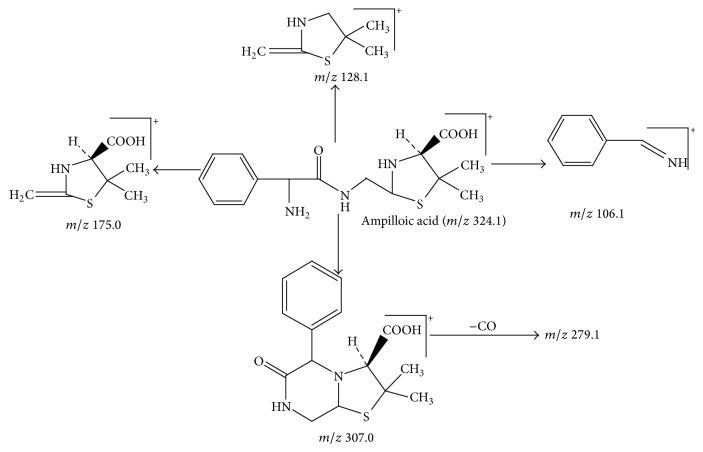
Proposed fragmentation pathway for the fragmentation ions of ampilloic acids.

Peak 4 gave a protonated molecular ion [M + H]^+^ with an *m*/*z* value of 324.1, major fragment ions at *m*/*z* 307.0, 279.1, 201.0, 175.0, 147.1, 128.1, and 106.1 in ESI^+^ mode. Peak 4 was tentatively identified as (5R) or (5S) ampilloic acid based on its characteristic ions (*m*/*z* 324.1, 307.0, 279.1, 128.1, and 106.1) and comparison with the published literature of known related substances of ampicillin [10]. Peak 6 produced major fragment ions at *m*/*z* 364.1 [M + K + H]^+^, 191.9, 174.0, 128.0, and 106.2. They all had similar MS fragmentation patterns (*m*/*z* 191.9, 174.0, 128.0, and 106.2). By comparison with the published literature [1], Peak 6 was tentatively identified as (5R) or (5S) ampilloic acid. (5S) or (5R) ampilloic acids were the isomers of ampilloic acids which were reported to be metabolites or degradation products of ampicillin [1]. However, the exact structure of these two components could not be determined due to the limited information.  Figure 6 showed the proposed MS fragmentation pathway for the fragmentation ions of ampilloic acids.

**Figure 7:**
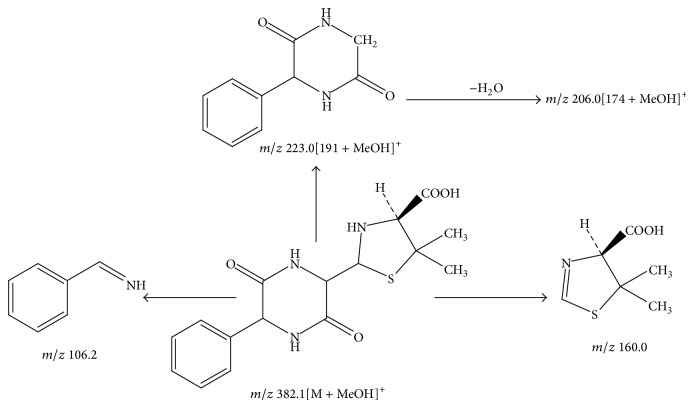
Proposed fragmentation pathway for the fragmentation ion of diketopiperazines of ampicillin.

Peak 7 had a molecular weight of 350 ([M + MeOH + H]^+^, *m*/*z* 382.1) and five major fragment ions were observed at *m*/*z* 331.1, 223.0 [191 + MeOH]^+^, 206.0 [174 + MeOH]^+^, 160.0, and 106.2. As it was reported [8], the two characteristic fragment ions *m*/*z* 160.0 and 106.2 were the representative fragment ions of ampicillin. By comparison with the published literature [7], this component was tentatively identified as diketopiperazines of ampicillin.  Figure 7 showed the proposed MS fragmentation pathway for the fragmentation ion of diketopiperazines of ampicillin.

**Figure 8:**
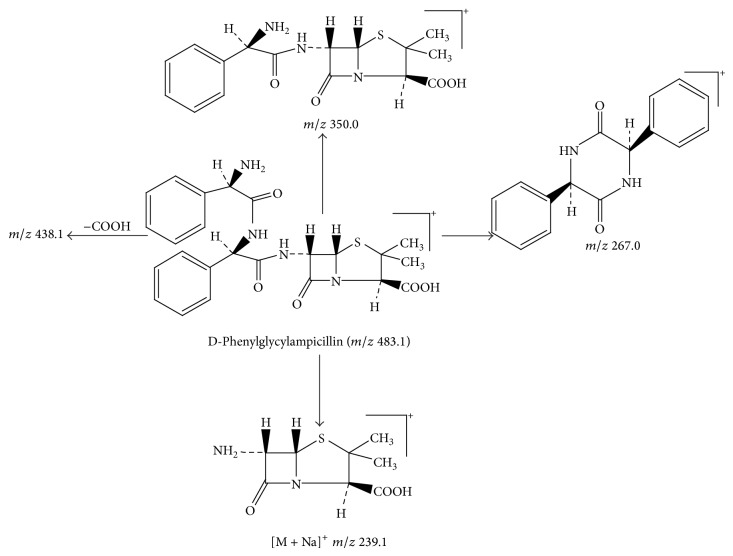
Proposed fragmentation pathway for the fragmentation ion of D-phenylglycylampicillin.

Peak 10 produced a protonated molecular ion at *m*/*z* 483.1 [M + H]^+^, the major fragment ions at *m*/*z* 439.1, 350.0, 267.0, and 239.1 in ESI^+^ mode. Fragment ion at *m*/*z* 439.1 could be attributed to loss of one −COOH from the ion (*m*/*z* 483.1). Based on the mass spectra, Peak 10 was identified as D-phenylglycylampicillin [1, 11].  Figure 8 showed the proposed MS fragmentation pathway for the fragmentation ion of D-phenylglycylampicillin.

Peak 15 had a molecular weight of *m*/*z* 699.1 [M + H]^+^ and three major fragment ions *m*/*z* 540.1, 381.1, and 248.0 in ESI^+^ mode. Upon collision-induced dissociation (CID), the ion at *m*/*z* 699.1 eliminated one molecule of thiazolidine ring to produce *m*/*z* 540.1. The *m*/*z* 540.1 ion could further lose one molecule of thiazolidine ring successively to give significant *m*/*z* 381.1 fragment ion. Thus, Peak 15 was identified as closed-cycle dimer based on the published literature [1]. Closed-cycle dimer was the main cause of allergy, so that we must control the amount of this related substance in ampicillin capsule.

Peak 16 produced major fragment molecular ions at *m*/*z* 524.8 [M + H]^+^, 889.3, 730.3, 571.2, and 160.0 in ESI^+^ mode. Upon CID, the ion at *m*/*z* 1048 [M]^+^ eliminated one molecule of thiazolidine ring to produce *m*/*z* 889.3. The *m*/*z* 889.3 ion could lose one molecule of thiazolidine ring successively to give significant *m*/*z* 730.3 ion. The *m*/*z* 730.3 ion could further lose one molecule of thiazolidine ring successively to give significant *m*/*z* 571.2. The fragment ion *m*/*z* 160.0 is characteristic thiazolidine ring of ampicillin. Thus, Peak 16 was identified as closed-cycle trimer based on the published literature [1]. Closed-cycle trimer was also the main cause of allergy, so that we must control the amount of this related substance in ampicillin capsule.

### 3.3. Identification of the Unknown Related Substances in Ampicillin Capsule

This part of the investigation was to identify the chemical structures of unknown related substances which were not yet reported in ampicillin capsule based on the mass fragment characterization and cleavage pathways of ampicillin and its known related substances. By means of the RRLC-ESI-MS^n^ experiments, in this part, chemical structures of four related substances were tentatively identified in ampicillin capsule for the first time.  Table 3 showed the chromatographic and mass spectral characteristics of the above unknown related substances detected by RRLC-ESI-MS^n^ in ampicillin capsule.

**Figure 9:**
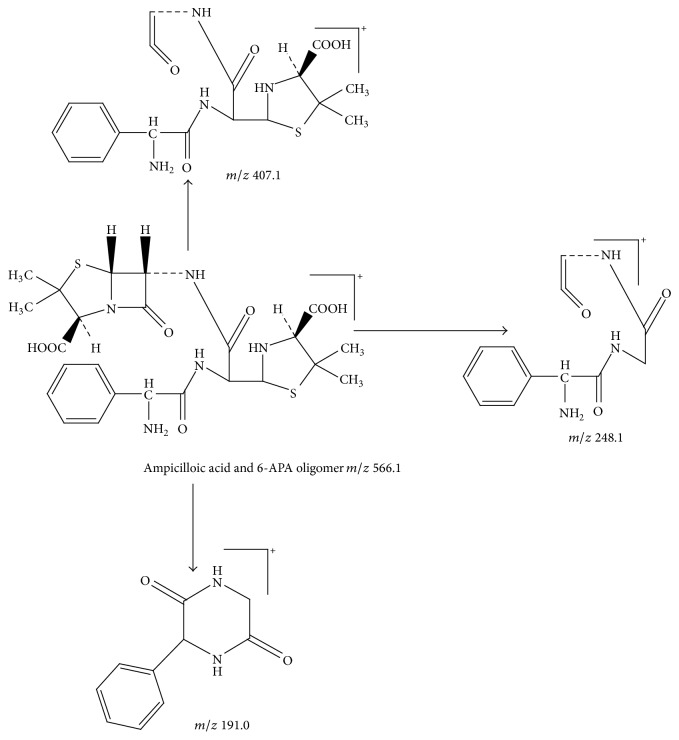
Proposed fragmentation pathway for the fragmentation ion of ampicilloic acid and 6-APA oligomer.

Peak 9 had a molecular weight of *m*/*z* 566.1 [M + H]^+^ and three major fragment ions *m*/*z* 407.1, 248.1, and 191.0 in ESI^+^ mode. The fragment ions at *m*/*z* 407.1 and 248.1 eliminated one molecule of thiazolidine ring successively from ion at *m*/*z* 566.1 [M + 1]^+^. The fragment ion at *m*/*z* 191.0 probably should be a characteristic fragment ion of ampicillin piperazine-2,5-dione [12]. Thus, Peak 9 was identified tentatively as ampicilloic acid and 6-aminopenicillanic acid (6-APA) oligomer.  Figure 9 showed the proposed MS fragmentation pathway for the fragmentation ion of ampicilloic acid and 6-APA oligomer.

**Figure 10:**
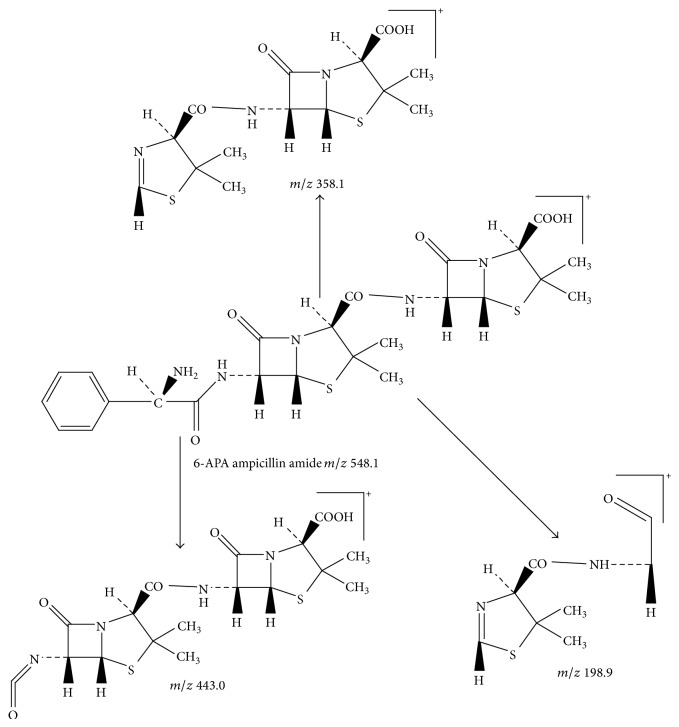
Proposed fragmentation pathway for the fragmentation ion of 6-APA ampicillin amide.

Peak 12 produced a protonated molecular ion at *m*/*z* 548.1 [M + H]^+^ and three major fragment ions at 443.1, 358.1, and 199.0. Based on fragment ions, Peak 12 was tentatively identified as 6-APA ampicillin amide.  Figure 10 showed the proposed MS fragmentation pathway for the fragmentation ion of 6-APA ampicillin amide.

**Figure 11:**
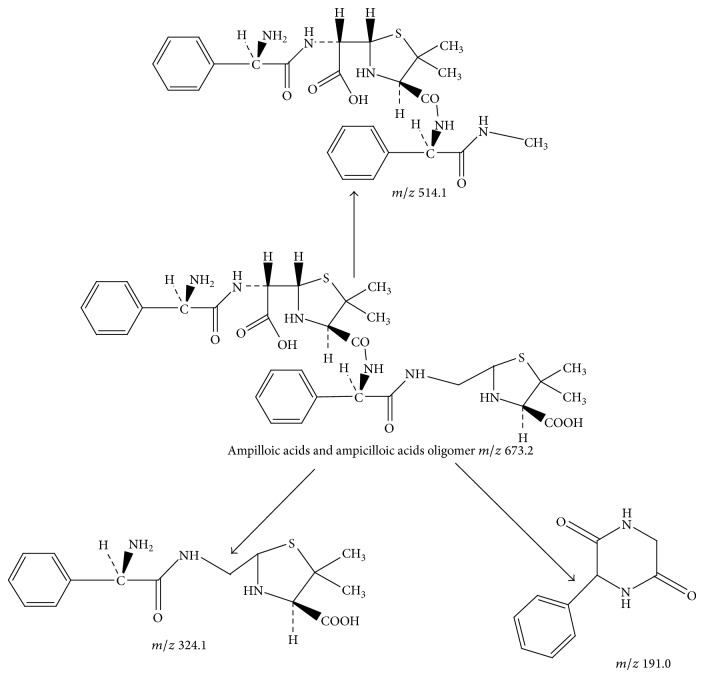
Proposed fragmentation pathway for the fragmentation ion of ampilloic acids and ampicilloic acids oligomer.

Peak 13 had a molecular weight of 673.2 [M + H]^+^ and four major fragment ions *m*/*z* 655.1, 514.1, 324.1, and 191.0. The fragment ions at *m*/*z* 655.1 and 514.1 were attributed to the loss of one water molecule (18 Da) and one molecule of thiazolidine ring from ion at *m*/*z* 673.2. The fragment ion at *m*/*z* 324.1 was molecular weight of ampilloic acids. The fragment ion at *m*/*z* 191.0 was the fragment ion of ampicilloic acids. Peak 13 was identified tentatively as ampilloic acids and ampicilloic acids oligomer.  Figure 11 showed the proposed MS fragmentation pathway for the fragmentation ion of ampilloic acids and ampicilloic acids oligomer.

Peak 14 produced a protonated molecular ion at *m*/*z* 483.1 [M + H]^+^, which was identified as the other isomer of D-phenylglycylampicillin because Peak 14 and Peak 10 both showed the same diagnostic ions at *m*/*z* 439.1, 350.1, 239.1, and 160.0.

## 4. Conclusion

The RRLC-ESI-MS^n^ technique was successfully established to rapidly determine and identify the structures of the related substances in ampicillin capsule. RRLC is efficient in separating chemical compounds in a mixture, and MS provides abundant information for structural elucidation of the compounds when tandem mass spectrometry is applied [13]. Although ampilloic acids, ampicilloic acid, and closed-cycle dimer had been investigated previously by LC-MS method, MS information and characteristic diagnostic ions of a number of components in ampicillin capsule were described simultaneously for the first time. None of the previously reported methods have led to so much chemical information on the related substances in ampicillin capsule. The results of this study had identified 13 out of 15 related substances in ampicillin capsule. Unfortunately, three groups of isomers (Peak 1 and Peak 2, Peak 4 and Peak 6, and Peak 10 and Peak 14) and condensation of amino and carboxyl groups (Peak 9, Peak 12, and Peak 13) could not be identified fully by current RRLC-ESI-MS^n^ information. Peak 8 and Peak 11 had not yet been identified based on current mass spectra information. In summary, this investigation had provided an example of the rapid identification of related substances in ampicillin capsule. The meaningful information for the related substances in ampicillin capsule could lead to the development of the understanding of the quality and safety of the drug.

## Figures and Tables

**Table 1:** The structures of the known related substances of ampicillin.

Number	Name of related substances	Chemical structure
1	6-Aminopenicillanic acid (6-APA)	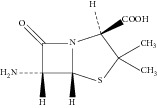

2	L-Ampicillin	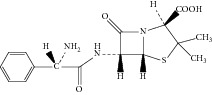

3	Diketopiperazines of ampicillin	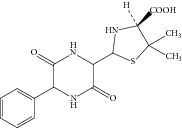

4	Ampicilloic acid	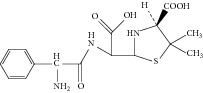

5	Ampilloic acid	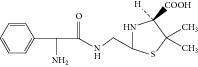

6	Ampicillinyl-D-phenylglycine	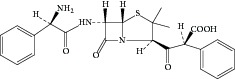

7	(3R,6R)3,6-Diphenylpiperazine-2,5-dione	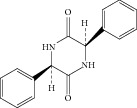

8	3-Phenylpyrazin-2-ol	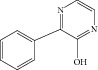

9	D-Phenylglycylampicillin	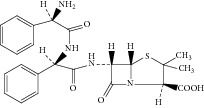

10	N-Pivaloyl-6-APA	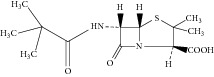

11	N-Pivaloylphenylglycine	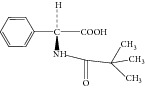

12	D-Phenylglycine	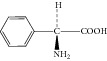

13	Open-cycle dimer	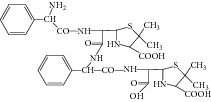

14	Closed-cycle dimer	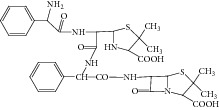

15	Open-cycle trimer	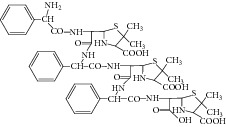

16	Closed-cycle trimer	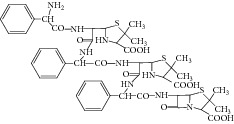

17	Open-cycle tetramer (*n* = 2)	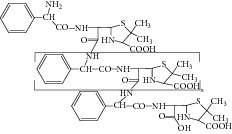

18	Closed-cycle tetramer (*n* = 2)	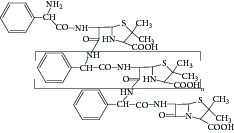

**Table 2:** Results of identification of the known related substances in ampicillin capsule.

Peak number	RT (min)	MS (*m*/*z*)	MS^2^ (*m*/*z*)	Identification
1	1.8	368.1 [M + H]^+^	324.1; 307.1; 279.2	(5S, 6R) ampicilloic acid
2	2.2	368.1 [M + H]^+^	324.1; 307.2; 279.2; 175.1	(5R, 6R) ampicilloic acid
3	2.5	350.1 [M + H]^+^	333.0; 192.0; 174.0; 160.0; 106.1	L-Ampicillin
4	2.8	324.1 [M + H]^+^	307.0; 279.1; 201.0; 175.0; 147.1; 128.1; 106.1	(5S) or (5R) ampilloic acids
5	4.2	350.1 [M + H]^+^	333.1; 192.0; 174.0; 159.9; 106.2	Ampicillin
6	8.1	364.1 [M + K^+^H]^+^	191.9; 174.0; 128.0; 106.2	(5S) or (5R) ampilloic acids
7	9.0	382.1 [M + MeOH]^+^	331.1; 223.0; 206.0; 160.0; 106.2	Diketopiperazines of ampicillin
10	12.3	483.1 [M + H]^+^	439.1; 350.0; 267.0; 239.1	D-Phenylglycylampicillin
15	17.0	699.1 [M + H]^+^	540.1; 381.1; 248.0	Closed-cycle dimer
16	19.3	524.8 [1/2M + H]^+^	889.3; 730.3; 571.2; 160.0	Closed-cycle trimer

**Table 3:** Results of identification of the unknown related substances in ampicillin capsule.

Peak number	RT (min)	MS (*m*/*z*)	MS^2^ (*m*/*z*)	Identification
9	11.1	566.1 [M + H]^+^	407.1; 248.1; 191.0	Ampicilloic acid and 6-APA oligomer
12	13.9	548.1 [M + H]^+^	443.1; 358.1; 199.0	6-APA ampicillin amide
13	15.2	673.2 [M + H]^+^	655.1; 514.1; 324.1; 191.0	Ampilloic acids and ampicilloic acids oligomer
14	15.5	483.1 [M + H]^+^	439.1; 350.1; 239.1; 160.0	Isomer of D-phenylglycyl ampicillin
